# Dr. Edgard San Juan: a feminine look

**DOI:** 10.5935/1678-9741.20150049

**Published:** 2015

**Authors:** Vera Lucia Amaral Molari Piccardi

**Affiliations:** 1 Specialist Member of the Brazilian Society of Cardiovascular Surgery. Title of specialist in cardiovascular surgery by the Brazilian Medical Association. Specialist Member of the Brazilian Society of Cardiology. Title of specialist in cardiology by the Brazilian Medical Association. Qualified member of the Department of Cardiac Pacing (DECA).Cardiovascular Surgeon of Real e Benemérita Associação Portuguesa de Beneficência, Pulmo Cor Pneumologia e Cardiologia Clínica e Cirúrgica, and Associação Beneficente e Filantrópica da Cruz Azul de São Paulo. São Paulo, SP, Brazil.

The first time I met Professor San Juan and his cardiothoracic surgeons was in 1976, when I started my medical residency in cardiovascular surgery at São Joaquim Hospital of the Real and Benemérita Associação Portuguesa de Beneficência de São Paulo.

At that time, I didn't have the pleasure of knowing him personally; my relationship was limited to his residents and assistants when we sometimes met one another in hospital wards or during shifts in the Intensive Care Unit, where we took care of patients after surgery.

Surgical procedures were performed for many years in the old operating theatres on the ninth floor of São Joaquim Hospital, numbers 29 and 31, separated by the washbasin. They were lit by large glazed windows with a beautiful view of the city of São Paulo, a real postcard appreciated during surgery breaks.

At this time, only three teams of cardiac surgery worked at Beneficência Portuguesa Hospital, distinguishing themselves by the pioneering of high complexity surgeries and boosting their development, with the use of cardiopulmonary bypass and artificial oxygenators: Professor Euryclides de Jesus Zerbini, Professor Adib Domingos Jatene and Professor Edgard Schroeder San Juan (Figure 1), whose this biography is.

**Fig. 1:**
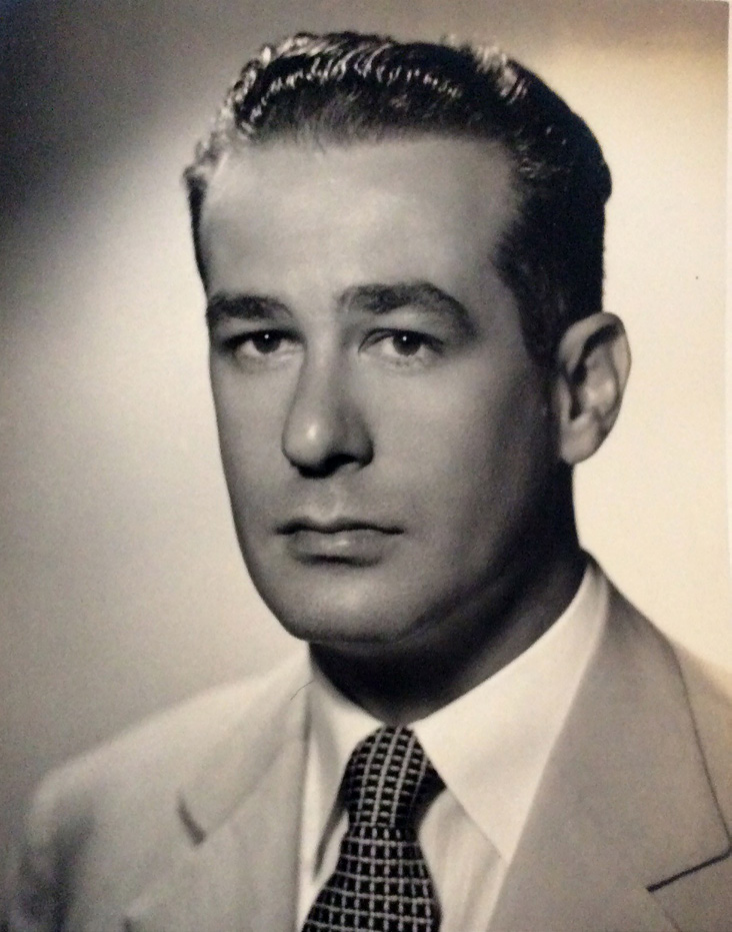
Professor Edgard Schoeder San Juan.

Professor San Juan was born in São Paulo, Campos Elísios, neighborhood, on February 23, 1919, descended from European immigrants. His father, Mr. Francisco Severo San Juan, was a civil engineer of Spanish descent from the city of Valencia; a very educated man, worked for Santos City Hall, São Paulo, having participated in the construction of São Vicente Suspension Bridge in the mid-twentieth century. He married Herminia Schroeder, a woman of German descent, who worked as an assistant at a dentist office. The couple had three children: Edgard, Odilon and Ondina.

The family lived for many years in Santa Cecilia, a neighborhood in São Paulo, on Tatuí Baron Street. The eldest son, Edgard, was dedicated to studies spending hours in the basement of the house reading and doing simple experiments. He was a very dedicated boy who liked to study a lot and soon showed his inclination to practice medicine and care for the sick.

Young Edgard attended Saint Adalbert Schule (Escola Santo Adalberto). In 1932, he enrolled in the Ginásio Normal graduating in 1936 and then joined the preparatory course for Medicine (pre-medical program), at Ginásio de São Bento until 1938, when he joined college.

He went to the University of São Paulo Medical School and graduated in 1944 (Figure 2).

**Fig. 2:**
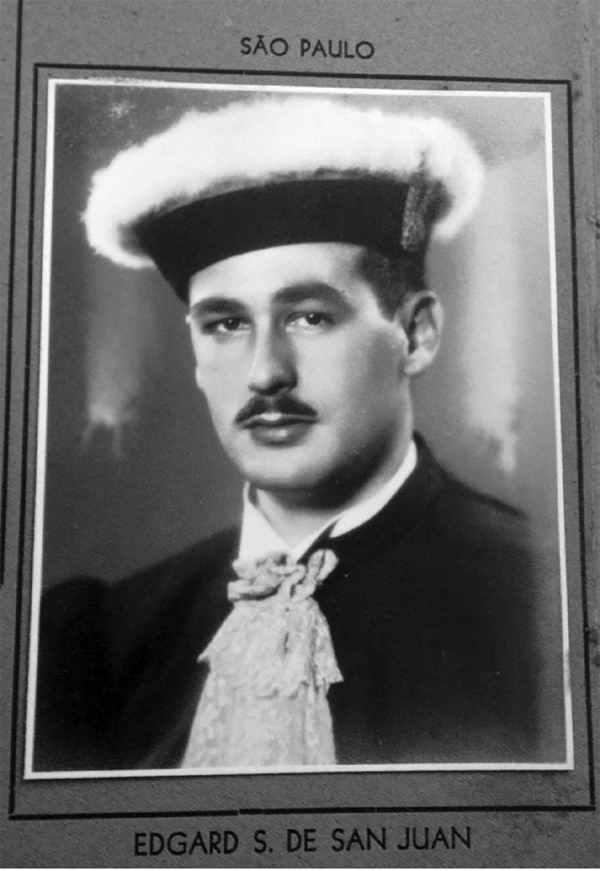
Graduating from the University of São Paulo Medical School in 1944.

He finished his doctorate degree in Medicine in December 1950 in the same college. Dr. Edgard presented a methodical and systematic study on the topographic distribution of elements and pulmonary pedicle, under the title "Anatomical Observation on Bronchial Distribution in Human's Middle Lobes of the Lung".

He became professor of Clinical Surgery at the University of São Paulo Medical School in 1954, presenting a thesis on "The Surgical Treatment of Congenital Lung Cysts".

During his studies, he expressed great interest in surgical procedures and found true love for surgery, which accompanied him throughout his life.

He was a disciple of Professor Zerbini in the early fifties and member of the "Thoracic Group" (Grupo de Tórax) of the first surgical clinic, accompanying him on surgical procedures at Santa Casa de Misericórdia de São Paulo and at the new Clinical Hospital (Hospital das Clínicas), recently opened in São Paulo (Figure 3). He stood out in lung, pleural, mediastinal surgery, and also in the surgical treatment of tuberculosis.

**Fig. 3:**
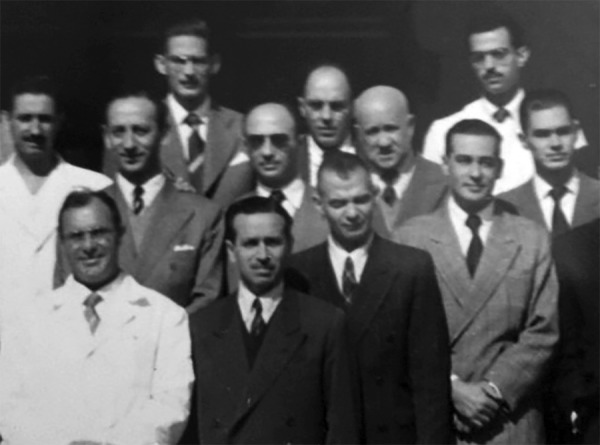
Thoracic Group - First Surgical Clinic (FMUSP).

However, engage in cardiac operations was his dream.

In the late forties, Dr. Edgard participated in pioneering surgeries, as treatments for coarctation of the aorta, as well as closure of patent ductus arteriosus, conventionally, which allowed the surgeons of that time overcome the technical difficulties of large blood vessel's anastomosis.

The experience and confidence acquired, encouraged them to carry out more complex cardiac surgeries, getting better results.

Shortly after, Professor San Juan performed his first mitral valve surgery: one Digital Mitral Commissurotomy, a technique initiated in the modern era by Bailey of Philadelphia, and Harken from Boston in 1948, with good results in the treatment of rheumatic mitral stenosis. He actively participated in the work carried out by Dr. Zerbini compiling 126 patients with mitral valve disease undergoing digital commissurotomy as from 1951.

Being a hardworking doctor, always interested in new techniques developed abroad, Dr. Edgard actively participated in the early days of the use of extracorporeal circulation in Brazil

In April 1957, he traveled to London, where attended a graduate program in Thoracic Surgery at the Institute of Diseases of Chest, watching the work of Professor Price Thomas, at Bromptom Hospital (Figure 4). He remained until November at Guy's Hospital, closely watching cardiac interventions with hypothermia aid and extracorporeal circulation performed by Dr. Brock.

**Fig. 4:**
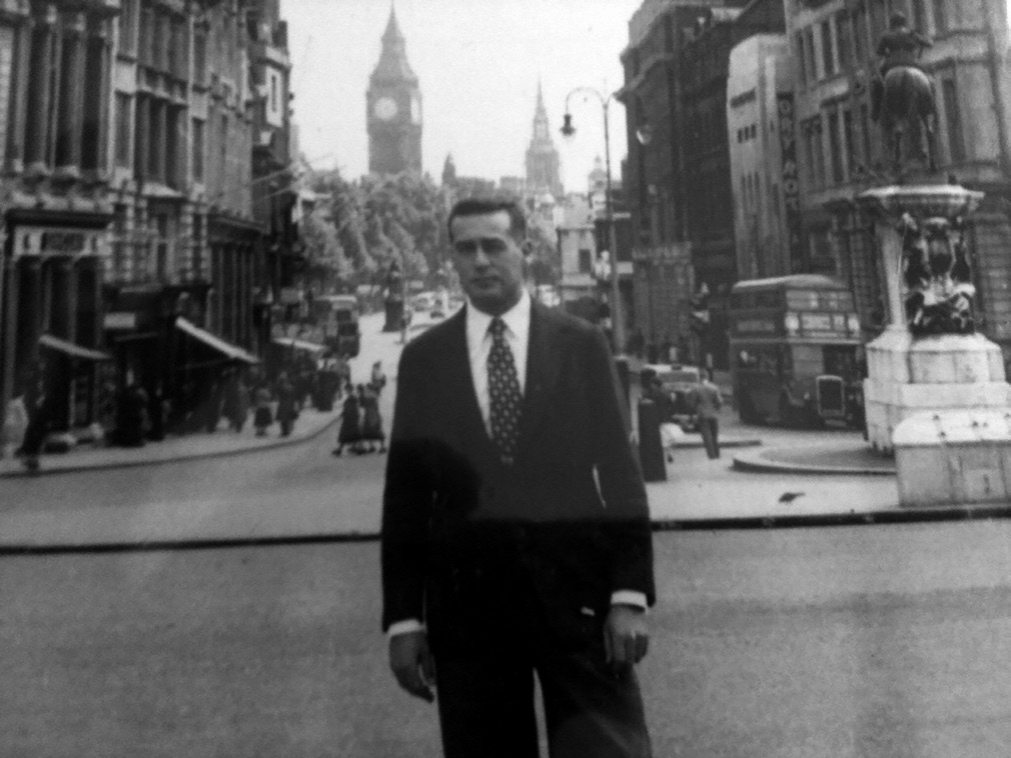
Professor San Juan in London, Master’s Degree in Thoracic Surgery-1957.

He returned to São Paulo convinced to perform heart surgeries using new techniques in his patients (Figure 5).

**Fig. 5:**
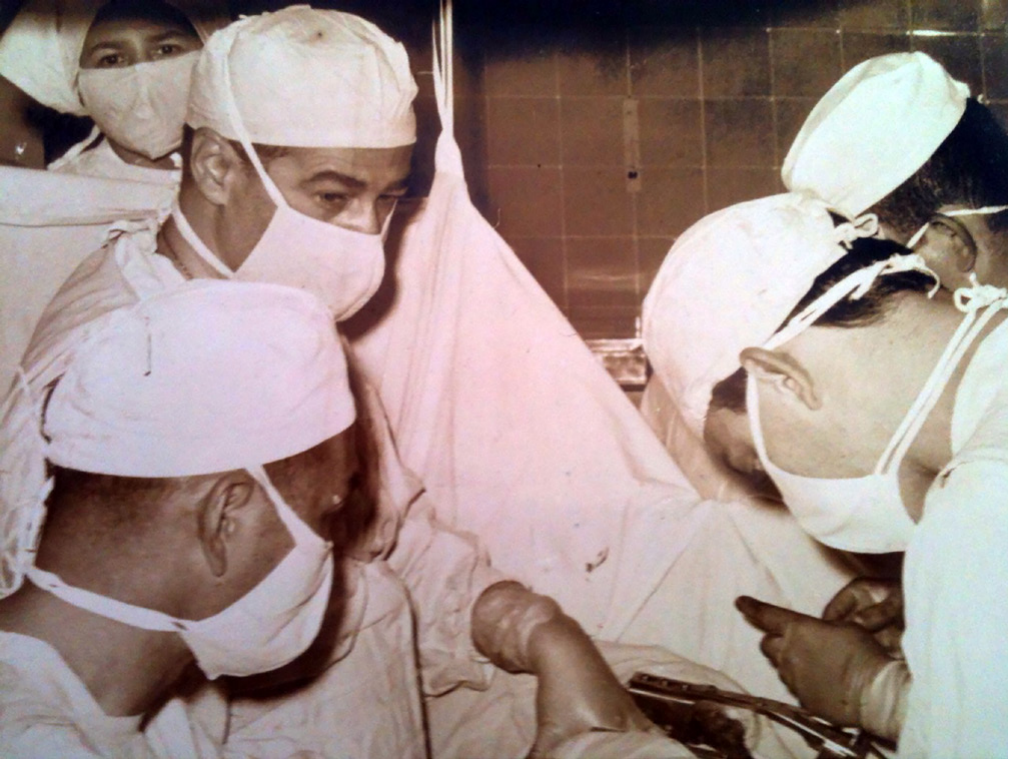
Professor San Juan in cardiac surgery at the Clinical Hospital – FMUSP.

On August 18, 1958, he participated with Professor Zerbini; in the first heart surgery with extracorporeal circulation at the "Hospital das Clinicas", it was a case of pulmonary valve stenosis successfully corrected.

This was the beginning of an embryonic cell, which created a new medical specialty, allowing surgical treatment of the heart with the opening of the cardiac cavities under direct vision.

This allowed surgeons to perform: 1- corrections, or replacement of diseased valves, 2- detailed vision of congenital defects to be corrected, and 3- myocardial revascularization through implantation of venous or arterial grafts.

He was the first disciple of Professor Zerbini to start his own team, becoming a full member of Cardiovascular and Pulmonary Surgery at Beneficência Portuguesa Hospital in São Paulo since 1958.

Professor San Juan finished his post doctorate degree in Thoracic Surgery at the Escola Paulista de Medicina in 1970 and in Cardiovascular Surgery at the Escola Nacional de Medicina, in 1972 in Rio de Janeiro, with the thesis entitled "Surgical Treatment of Atrio-Ventricular block performed by pacemaker Implant".

He returned to London in September 1970 to take the Cardiac Surgery Course at the National Heart Hospital (Figure 6). In 1971, Professor San Juan went to the United States to do an internship at Cleveland Clinic in the Department of Cardiovascular Surgery and in 1974 returned to the US for an internship program at the Hospital for Sick Children in Buffalo and St. Luke's and Texas Children's Hospital, in Houston, under the supervision of Dr. Denton A. Cooley.

**Fig. 6:**
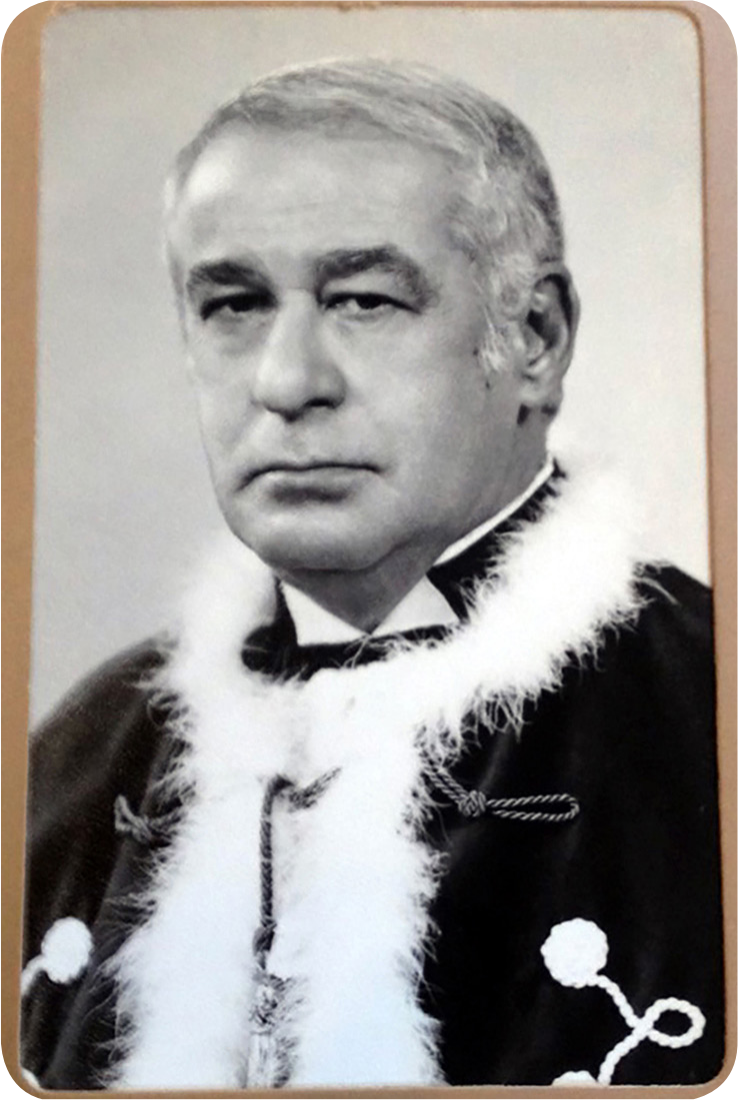
Professor Edgard San Juan - Post Doctorate Degree in 1970.

He returned to Brazil bringing expertise in the treatment of congenital heart diseases, particularly related to Tetralogy of Fallot, his favorite congenital disease.

As he had didactic teaching methods, Dr. Edgard was a full Professor of Cardiovascular and Pulmonary Surgery at Bragança Paulista Medical School, from 1975 to 1985. He could combine really well his surgical and educational activities, teaching his classes eloquently in order to pass on medical knowledge to his students.

In 1975, he joined other prominent surgeons in São Paulo, such as Dr. Emil Sabino, his assistant and partner for many years, founding the "Pulmo-Cor Edgard San Juan e Médicos Associados" (Figure 7).

**Fig. 7:**
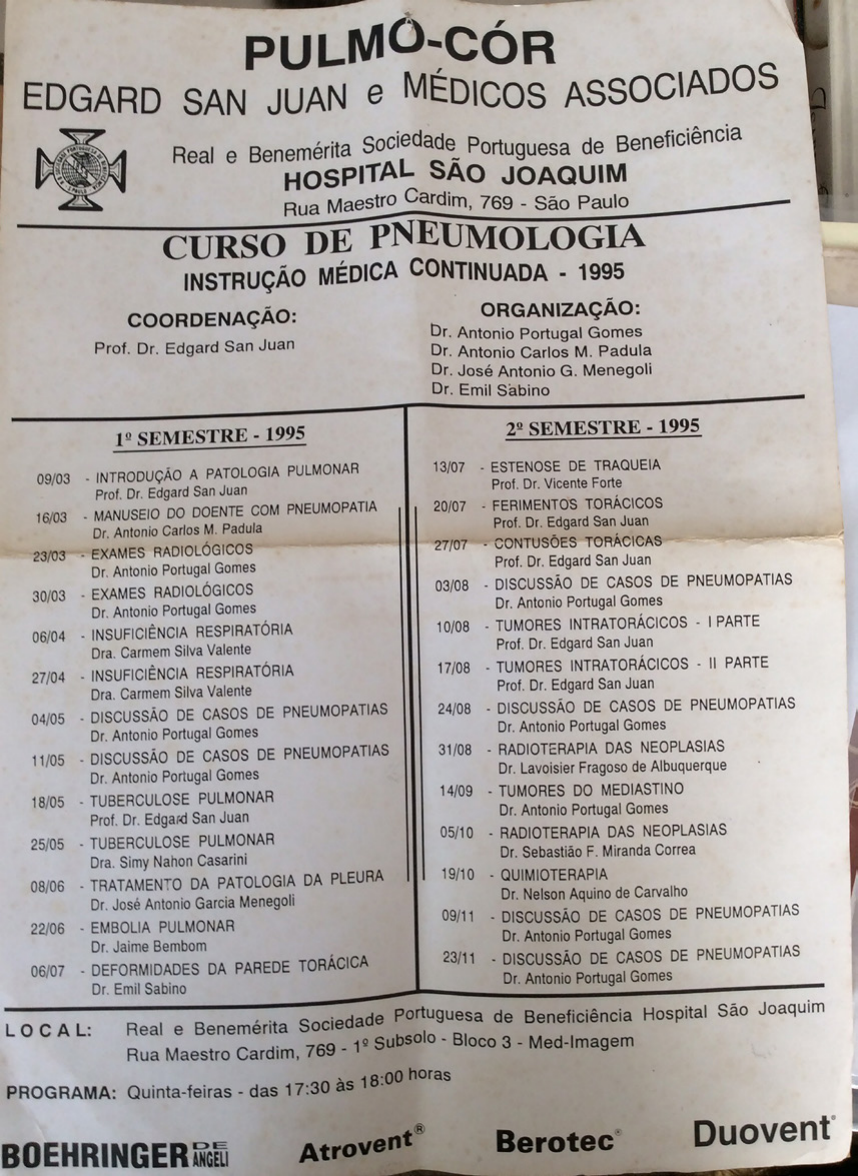
Pulmo-Cor - Pneumology Course Summary.

The clinic was aimed at medical care in Cardiology and Pneumology in clinical and surgical areas as well, Medical Residency and Specialization programs. Their Residents were from not only other states of Brazil, but from other countries like Central America and South America, such as Honduras, Dominican Republic, Bolivia and others. Many of these students have become renowned and prestigious international professionals in the medical field.

He devoted himself intensely to assistential medicine, having operated a great number of patients, which represented the substrate, to write numerous medical books, and publish many very important articles of high interest mainly in the surgical area.

With his great administrative skills, Dr. San Juan was the leader of Cardiac and Pulmonary Surgery at the "Hospital do Servidor Público Estadual de São Paulo", IAMSPE, between 1963 and 1978.

He was the doctor of distinguished people, such as Vinicius de Morais and Marcondes Filho, the Minister of Finance of Getúlio Vargas administration. Years earlier, received Princess Lilian of Belgium, who watched a cardiac surgery with extracorporeal circulation in 1962 at the "Hospital das Clínicas", University of São Paulo Medical School (Figure 8).

**Fig. 8:**
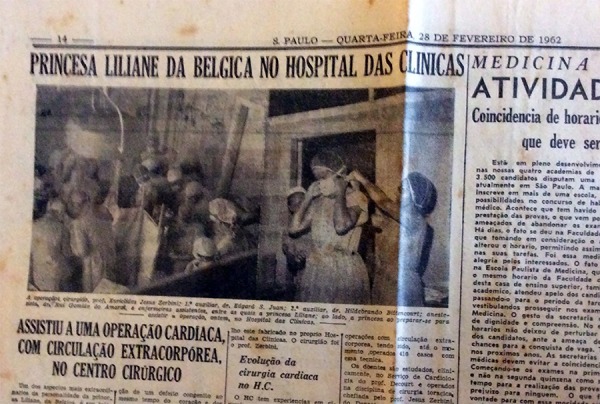
Princess Lilian of Belgium visiting the Hospital das Clínicas (Clinical Hospital) – FMUSP.

He was a distinguished Fellow, of the American College of Surgeons.

In Brazil actively participated at various medical societies: founding partner of the "Sociedade Brasileira de Cirurgia Cardiovascular". Full member at "Academia Paulista de Medicina", Full Member of "Colégio Brasileiro de Cirurgiões" Chapter São Paulo.

Full member of the "Sociedade Brasileira de Cirurgia Cardiovascular," where he held several position in the Board of Directors. Full member at Brazilian Society of Cardiology, member of the Department of Pediatrics, and also Brazilian Society of Pulmonology and the Brazilian Society of Thoracic Surgery.

Professor San Juan was devoted to gardening, in his leisure time. He was always interested with his plants and flowers, which surround the beautiful house where he lived, in Morumbi.

With eclectic tastes, listening to music was his other hobby. His musical taste would go from classical music until Frank Sinatra and Michael Jackson.

He was also devoted to his family in his little free time, sometimes as a very understanding father and friend for all times, or as wise and experienced counselor of his five children (Figure 9).

**Fig. 9:**
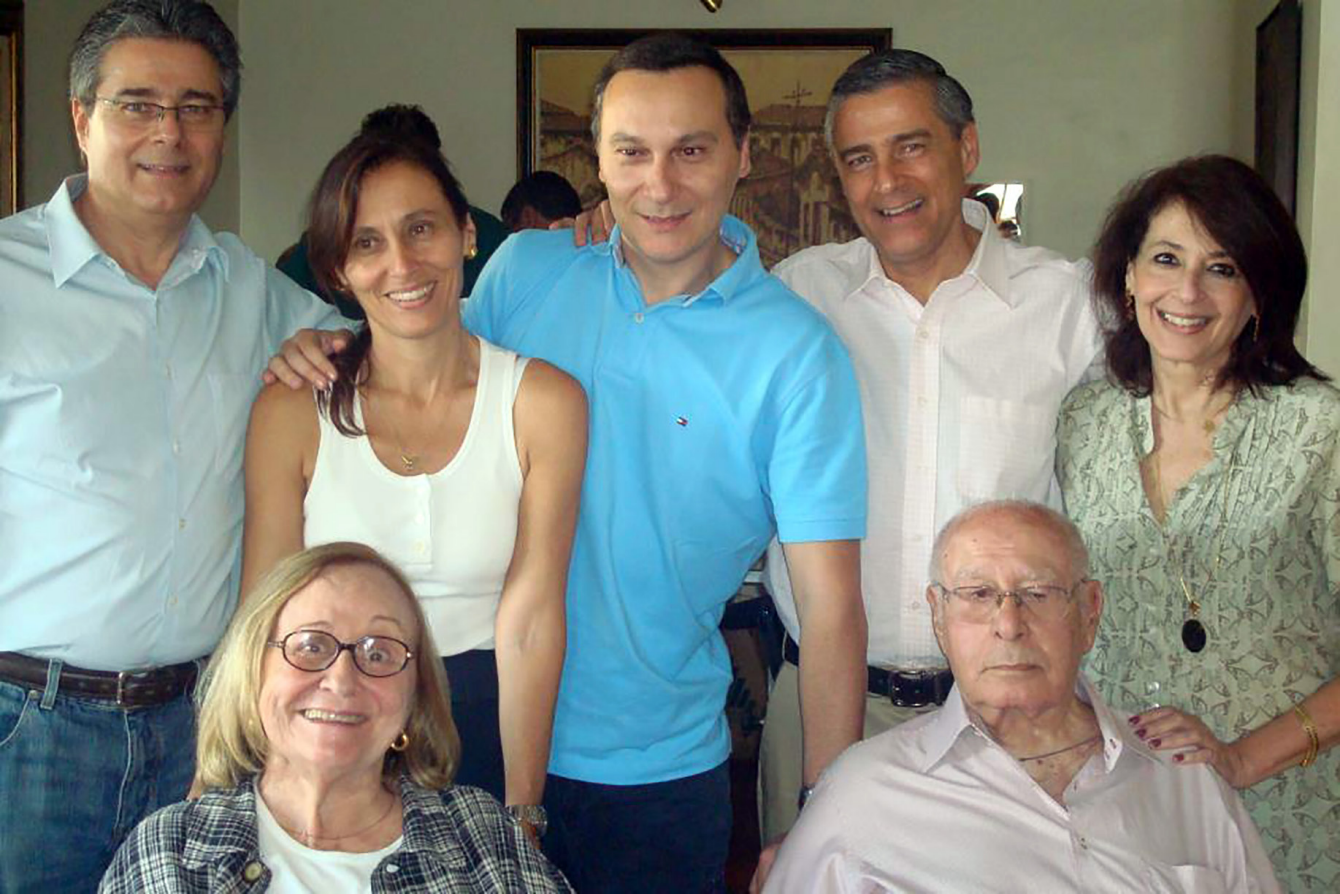
Professor San Juan accompanied by his children.

I personally met Professor San Juan in 1993 when I was invited to join the team as a surgeon. There was a pressing need for renewal of their surgical staff.

In his private clinic, I found a septuagenarian, but with traces of beauty and elegance, with a strong personality, which hid a generous and welcoming heart, an extremely organized and proud administrator admired by all his employees (Figure 10). After a few moments I felt at ease and we began a sincere friendship.

**Fig. 10:**
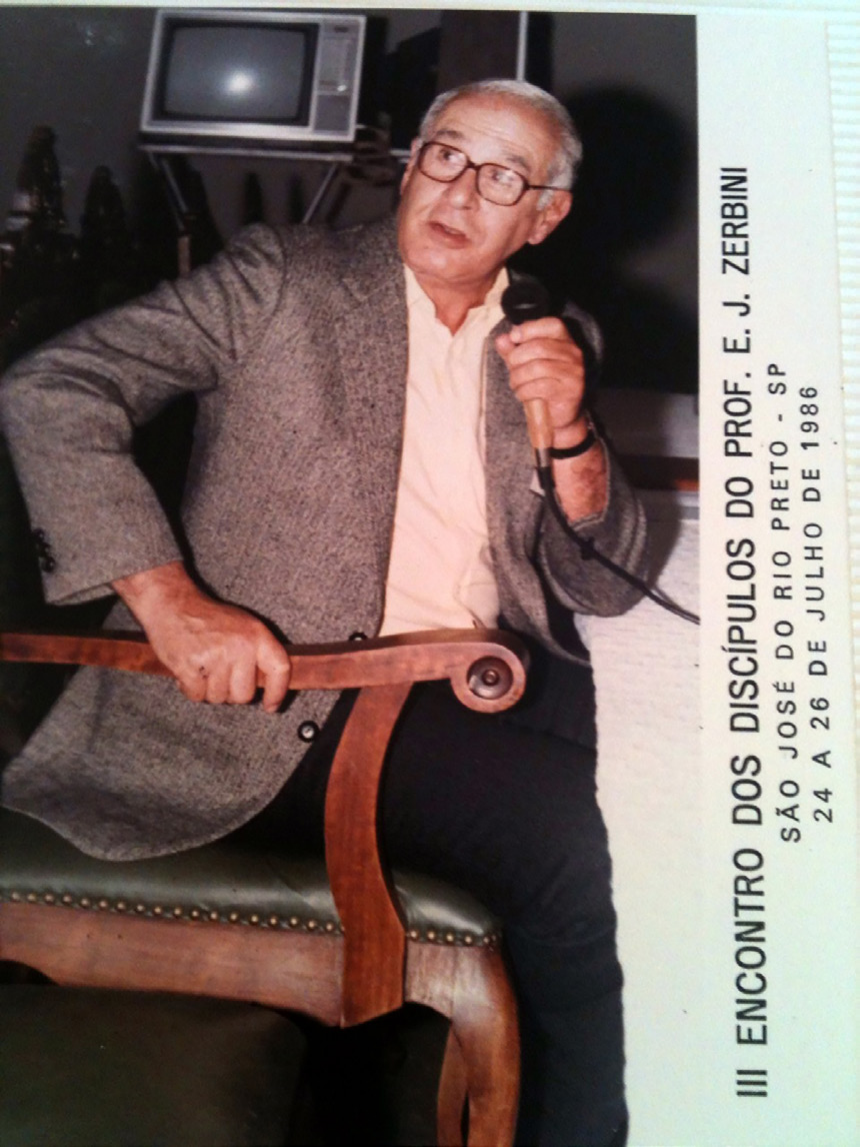
Professor San Juan - III Meeting of Professor Zerbini disciples.

He was surprised with no signs of bias, after all that would be a woman operating on his patients in the operating room!

He died on February 21, 2015, lucid and convinced that his mission had been accomplished at nearly 96 years old, always loving life and medicine.

Professor San Juan leaves a beautiful family with five children: Margarida San Juan Rozzino (Visual Artist); Edgard José San Juan (Economist); José Eduardo San Juan (Business Administrator); Flavia San Juan Laragnoit (Civil Engineer) and Cesar San Juan (Business Administrator), 16 grandchildren and 4 great-grandchildren (Figure 11).

**Fig. 11:**
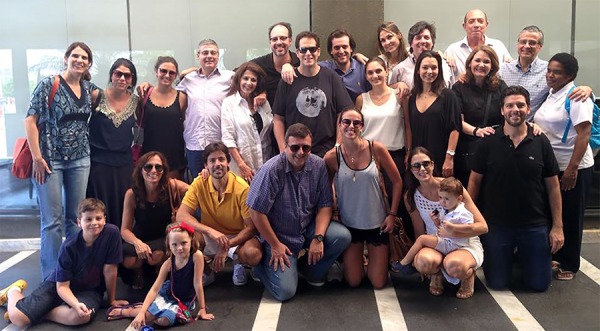
The Family of Professor Edgard San Juan.

Dr. Edgard leaves the Brazilian Cardiac Surgery a priceless legacy.

Professor Edgard Schroeder San Juan 1919 - 2015

## Messages from his children:

**Marguerite San Juan Rozzino**

"I would say that my father was a very charismatic and handsome man.

A great friend and confidant. He taught me the meaning of the words character and responsibility.

I'm very proud to be his daughter".

**Flavia San Juan Laragnoit**

"My father was a very intense person and lived his life to the fullest.

What to say about a person who lived so much and experienced so many things?

He was introspective and serious but at the same time liberal and ahead of his time.

A smart and loving man. He loved medicine.

He was also fond of his glass of red wine, sunbathing and simple life".

**Cesar San Juan**

"The memories I've had of my father since I was a child is that he was always studying, working in the hospital or business trips and/or medical conference ...

At first, I didn't understand this situation because I often missed him. However, I realized over time the importance of the profession that my father had chosen.

Besides, he used to do everything with love and dedication, working hard up to the day he could".

